# Supplementary material for: Multistage Psychometric Testing of the Homeless Health Access to Care Tool
Source: Int J Environ Res Public Health. 2022 Nov 29;19(23):15928. doi: 10.3390/ijerph192315928 (PMC9738957; doi:10.3390/ijerph192315928)
Supplement: Supplementary file 1 [file ijerph-19-15928-s001.zip › File S1 Instinctual Survey.pdf]

Instinctual Assessment of Case Studies of People Experiencing Homelessness

1. Introduction

Thank you for participating in this survey. You have been invited to participate in this survey because you have specific expertise and experience in providing care and support to people experiencing homelessness. Participation will involve completion of this electronic survey, which seeks your expert opinion on the level of vulnerability of 18 short case studies of people experiencing homelessness. We would like you to assess each case study as either highly vulnerable, moderately vulnerable or slightly vulnerable. We believe this will take around 10-minutes to complete. Below we provide definitions of vulnerability in relation to a person's health needs and their capacity to access healthcare. Please use these definitions when making your assessment.

By completing this survey you will be helping us to test the scoring system of a novel tool we have developed to assess the health need and the capacity of people experiencing homelessness to access healthcare. We hope to use this tool to prioritise people experiencing homelessness for healthcare and improve their access to healthcare.

Consent to participate in this study is deemed by completion of the survey. If you have any questions, please contact Dr Jane Currie at jane.currie@qut.edu.au

Definitions
Highly vulnerable – has multiple chronic, complex and or acute health needs and health is likely to deteriorate without urgent intervention. Will need one or more health care workers to support them to access healthcare (e.g. someone to transport them to appointments and stay with them throughout the consultation).

Moderately vulnerable – has several chronic, complex and or acute health needs and health might deteriorate without intervention. Will need verbal or written support to access healthcare and one on one support for some components of accessing healthcare (e.g. someone to stay with them during a healthcare consultation).

Slightly vulnerable – has some chronic, complex and or acute health needs and health is unlikely to deteriorate without intervention. Will need some guidance in terms of verbal and written support to access healthcare (e.g. written or verbal information on how to make an appointment, and can access the consultation on their own).

* 1. Please read the case study below, and based on your instinct of what you have read, assess the case study as either highly vulnerable, moderately vulnerable or slightly vulnerable.

Case 1 Summary

Ella is a 56 year old who identifies as female, originally from Iraq, arrived in Sydney 3-months ago to seek asylum. Ella speaks very little English and the support of an interpreter is required. Ella has been sleeping on her friend’s couch in a house in Newtown since her arrival. She doesn’t know how long she will be able to stay there, as her friend’s husband doesn’t like her staying there. She has no income at the moment and says she has no money. She has a painful lump in her left axilla, which she first noticed two months. The lump has grown bigger and more painful over time. Ella has a history of diabetes mellitus, she was diagnosed four years ago and is well controlled on Metformin 500mg BD. She only has two tablets left. She is regularly tearful because she thinks often of her marriage in Iraq, which was abusive, and of her children that are still living in Iraq. Ella shows signs of a depressed mood. Ella's friend told her she is worried about Ella's mental health. Ella has experienced depression but hasn’t taken medication for it. She has no thoughts of suicide or self-harm. She appears clean and tidy in her appearance. She has a mobile phone, and her friend is her emergency contact. She doesn’t smoke or drink alcohol or use drugs. She has no Medicare card and hasn’t seen a doctor for over a year. Her husband in Iraq prevented her from seeing a doctor and she is worried she cannot afford healthcare in Australia and therefore may not be able to access healthcare when feeling unwell.

- ◯ Highly vulnerable
- ◯ Moderately vulnerable
- ◯ Slightly vulnerable

Please provide the main rationale for your assessment decision

* 2. Please read the case study below, and based on your instinct of what you have read, assess the case study as either highly vulnerable, moderately vulnerable or slightly vulnerable.

Case 2 Summary

Debbie is a 19 year old who identifies as female, originally from Dubbo, she is experiencing per vaginal bleeding. Debbie had unprotected sex with someone other than her partner two days ago and received the morning after pill via a GP yesterday afternoon. She started to experience moderate per vaginal bleeding overnight and through this morning. Debbie appears dishevelled, clothes, skin and hair are dirty, she is trembly, tearful, has poor eye contact, mumbling speech and laughs nervously. Debbie has experienced depression and anxiety for a few years, and she was commenced on Lexapro 10mg (Escitalopram) but hasn't taken it for 6-months or so, as they were stolen. Debbie has some self-harm scars, approximately 2 weeks old on her upper arms. She isn't suicidal but has regular thoughts of self-harm. She was last in hospital six weeks ago after her boyfriend hit her about the head, and she became unconscious. She was admitted overnight and then discharged. She has had two other attendances to the local emergency department in the last 6-months with injuries from her boyfriend. Debbie worries about her mental health. She is using heroin regularly and her neighbour gives her the heroin for free, as does her boyfriend. She wants to stop using heroin but doesn't know how. She has been living in a squat with her boyfriend for the past 8-months. Debbie appears to be under the influence of an illicit substance, and she admits to using heroin this morning. She is fidgety and looks at her watch regularly because she has to go and meet her boyfriend soon or she'll be in trouble. She has a Medicare card, but has no income; she does have a mobile phone. Her boyfriend is her emergency contact.

- ○ Highly vulnerable
- ○ Moderately vulnerable
- ○ Slightly vulnerable

Please provide the main rationale for your assessment decision

* 3. Please read the case study below, and based on your instinct of what you have read, assess the case study as either highly vulnerable, moderately vulnerable or slightly vulnerable.

Case 3 Summary

Zihan, is a 50-year old who identifies as female, she has pain in her head, neck, ear, pain to her arms, and dizziness, secondary to an assault from her husband. Zihan speaks only Mandarin and an interpreter is required. Zihan moved to Sydney from China 6-months ago to marry her husband, who she met via the internet. She fled the marriage 3 days ago, as it had become extremely violent, with regular physical and sexual assaults. On the day she left her home, her husband had punched her to the head and attempted to strangle her, he told her he was going to kill her, so she ran away. She was able to escape and spent a night on the streets before accessing a room in a women's refuge, where she can now stay for 1-month. Zihan appears clean and tidy, she makes good eye contact and verbalises effectively via the telephone translator. She has no history of alcohol and other drug issues and no significant past medical history and no regular medications. She has no access to Medicare. She hasn't seen a health professional for over a year, but is capable of seeking healthcare. She has a mobile phone, and her emergency contact is a friend in Melbourne. She feels very anxious about what has happened to her and occasionaly has nightmares, reliving the assaults by her husband. She is scared that her husband might find her. She is also concerned about her own mental health. She has no thoughts of self-harm or suicide. She has no income.

- ◯ Highly vulnerable
- ◯ Moderately vulnerable
- ◯ Slightly vulnerable

Please provide the main rationale for your assessment decision

* 4. Please read the case study below, and based on your instinct of what you have read, assess the case study as either highly vulnerable, moderately vulnerable or slightly vulnerable.

Case 4 Summary

Asha is 21-year old who identifies as female, and she is worried because she cannot sleep at night. She goes to bed at around 10pm and when she lays down to rest, thoughts crowd her mind, and she cannot sleep. Asha is an Australian citizen, she fled her arranged marriage in India three years ago, she has a young daughter in Australia who her mother currently looks after. She and her mother are estranged. Her greatest fear is that she won't get to see her daughter again. Asha is difficult to engage in conversation, she appears to have a flat affect, shows very little emotion, and she answers questions slowly. Asha is rather hypervigilant to loud noises and the presence of other people and she is worried about her mental health. She appears clean and tidy. Asha was admitted to hospital a week ago for a mental health assessment secondary to suicidal thoughts and feelings of depression. She was commenced on Mirtazapine (30mg), which she takes as prescribed. She denies any suicidal thoughts at the moment but does have them regularly and also thinks of harming herself. Whilst in hospital she was diagnosed with a fatty liver, anxiety and post-traumatic stress disorder. Asha has sought healthcare via the emergency department on three occasions in the last 6-months. On discharge, Asha found temporary accommodation in a women's refuge. Prior to living in the refuge she had been living on the streets for around 3-months. She has found it difficult to hold down a job due to her mental health concerns. She has some money for medications, but no income. She drinks alcohol, around a bottle of wine a night to help her sleep. Asha denies any illicit drug use. She has a Medicare card. She has a mobile phone, but she doesn't have any friends or family that can be contacted in an emergency. Asha denies being pregnant.

- ○ Highly vulnerable
- ○ Moderately vulnerable
- ○ Slightly vulnerable

Please provide the main rationale for your assessment decision

* 5. Please read the case study below, and based on your instinct of what you have read, assess the case study as either highly vulnerable, moderately vulnerable or slightly vulnerable.

Case 5 Summary

Jessica is a 45 year old who identifies as female. Jessica self-discharged yesterday from Tierney House after a four day stay. She was admitted to hospital a week ago with a wound to her forearm, secondary to a domestic violence incident. Jessica had left her husband to live on the streets and he located her in Sydney CBD and they rowed, and he assaulted Jessica. She has had three hospital admissions in the past six-months for hyperglycaemia and was bought in by ambulance on two of those occasions. She has a past medical history of bipolar disorder, depression, Type I diabetes mellitus and hysterectomy. Her medications include Quetiapine and Insulin, both of which she takes only intermittently when she remembers. She wants to kill herself and has made ten attempts to do so in her life, her most recent attempt was last week. Jessica is constantly worried about her mental health. Jessica often does not seek healthcare when she needs to. She is well groomed, appears anorexic and is vague in her communication style. She appears to have difficulty concentrating and asks repeated questions about the same topic. She has had no investigations for cognitive impairment. She has an unsteady walking gait. Jessica has daily ICE use and uses heroin twice weekly. Jessica has income from Centrelink, she has a Medicare card, she lives with her husband, but leaves regularly when he is abusive. When she leaves her husband she either sleeps on a friends couch or on the streets. The longest time she has lived on the streets is for 7 months. Jessica provides her husband as her emergency contact.

- ◯ Highly vulnerable
- ◯ Moderately vulnerable
- ◯ Slightly vulnerable

Please provide the main rationale for your assessment decision

* 6. Please read the case study below, and based on your instinct of what you have read, assess the case study as either highly vulnerable, moderately vulnerable or slightly vulnerable.

Case 6 Summary

Sally is a 45 year old who identifies as female, and as a lesbian. Sally identifies as Aboriginal and her Aunty was part of the stolen generation. She presents with swelling to her lower legs and a lump to her left breast. Currently living in a women's refuge, Sally is on a waiting list for a permanent housing apartment. Sally appears clean and tidy, she wears clean clothes. Sally appears very drowsy and slightly unsteady on her feet. Sally has an engaging communication style but tends to fall asleep whilst speaking, which she says is because of the Methadone. She had been living on the streets for 2 years prior to moving into the refuge. Sally has an income from Centrelink, and she has a Medicare card. Sally has a previous addiction to heroin, she denies any current drug or alcohol use, but she appears very drowsy as if she is under the influence of an illicit substance. She smokes 20 cigarettes a day. Sally has bipolar disorder and hypertension. Current medications include Methadone, Lithium, and Ramipril, which she takes regularly now she is in the refuge. Sally isn't pregnant. Sally has seen a GP a week ago as she needed a repeat prescription, but she hasn't seen a GP for six months prior, or been to hospital. She doesn't trust healthcare professionals easily and tends to avoid seeking care until she is extremely unwell, or when she runs out of medication. She has no suicidal thoughts or thoughts of self-harm. She has a close friend as next of kin, and she has provided her mobile phone number.

- ○ Highly vulnerable
- ○ Moderately vulnerable
- ○ Slightly vulnerable

Please provide the main rationale for your assessment decision

* 7. Please read the case study below, and based on your instinct of what you have read, assess the case study as either highly vulnerable, moderately vulnerable or slightly vulnerable.

Case 7 Summary

Chandler is a 26 year old, who identifies as male and bisexual. Chandler identifies as Aboriginal and is not part of the stolen generation. Chandler is under the influence of drugs, possibly ICE and is finding it difficult to stand up and he can barely communicate his needs. He is leant against the wall in the Emergency Department waiting room. Chandler appears to be experiencing auditory hallucinations. He has a history of gastric ulcers, a fatty liver, a traumatic brain injury sustained 2 years ago from an assault, and schizophrenia, which was diagnosed 5 years ago. Chandler is holding his stomach and states that he has the worst abdominal pain he has ever experienced. Chandler appears dishevelled, his clothes are soiled, he is unshaven, and smells of alcohol and cigarette smoke. He has been sleeping on the streets for the past two months. Prior to that he was living with his girlfriend, but they recently broke up. Chandler has income from Centrelink, and he has access to Medicare but has lost his card yesterday when it was stolen along with his money and his phone. There is no way of contacting Chandler directly, but he goes to Wayside Chapel regularly. Chandler's medications include Olanzapine and Esomeprazole, which he often forgets to take. He keeps his medications in his pocket. Chandler has had over six presentations to the emergency department in the last four months, two of these via Ambulance. Chandler is able to seek healthcare when he needs to. A month ago he was admitted overnight following a collapse in the street secondary to drugs and alcohol. Chandler does not have thoughts of self-harm or suicide, but he is worried about his own mental health. Chandler's emergency contact is his ex-girlfriend.

- ◯ Highly vulnerable
- ◯ Moderately vulnerable
- ◯ Slightly vulnerable

Please provide the main rationale for your assessment decision

* 8. Please read the case study below, and based on your instinct of what you have read, assess the case study as either highly vulnerable, moderately vulnerable or slightly vulnerable.

Case 8 Summary

Jack is a 58 year old, who identifies as male. He fell over earlier in the day and has a minor wound on his left knee and a tear in his jeans. He is sat in a doorway, resting up against the wall. His speech is slurred and his breath smells of alcohol. He has been drinking heavily today and has consumed at least two litres of port. Jack has a dishevelled appearance, and his clothes are heavily soiled with faeces and urine and he is unshaven. Jack is very unstable on his feet and cannot safely mobilise unless he is leaning against a wall. Jack has a Medicare card, and gets some income from Centrelink. Jack has a past medical history of hypertension, gastric ulcers, liver cirrhosis, hepatitis C (currently untreated), and is being investigated for alcoholic dementia. Jack has his medications with him in a paper bag, they include Amlodipine and Esomeprazole, but he rarely remembers to take them. Jack was admitted to hospital two months ago and has been reviewed in the Emergency Department six times this month, two attendances were via Ambulance. On these two occasions a member of the public called the Ambulance, as Jack had collapsed in the street. Jack rarely seeks healthcare when he needs it. Jack denies any thoughts of self-harm or suicide and is not worried about his mental health. Jack doesn't have a phone, but he sleeps in this same doorway most nights and has done for the past seven years. He has a son in Sydney and gives his number as an emergency contact.

- ◯ Highly vulnerable
- ◯ Moderately vulnerable
- ◯ Slightly vulnerable

Please provide the main rationale for your assessment decision

* 9. Please read the case study below, and based on your instinct of what you have read, assess the case study as either highly vulnerable, moderately vulnerable or slightly vulnerable.

Case 9 Summary

Thomas is a 65 year old who identifies as male. Thomas has been sleeping on a train for the past few weeks. Prior to that he was sleeping in his car for around 12-months, until the Police requested that he move on, following complaints from the local residents. The Police were concerned that Thomas was experiencing auditory hallucinations and he became quickly aggressive with them when they asked if he was feeling OK. The Police took Thomas to hospital where he was admitted and diagnosed with schizophrenia. On dishcarge a week ago, he was been placed on a community treatment order (Olanzapine depot). Thomas doesn’t believe he is unwell and he doesn’t understand why he was admitted or why he needs medications. Thomas has been living off an inheritance, he is not currently working. He has history of hypertension and was prescribed Ramipril two years ago, but doesn't take the medication as he doesn't think he needs it. Thomas doesn't seek healthcare. He was diagnosed with depression as a teenager but never commenced on any medication. He denies any drug and alcohol use. He has no suicidal thoughts or thoughts of self-harm. Thomas appears to have a low mood, and occasionally appears preoccupied as if he is experiencing auditory hallucinations. Thomas has a clean appearance. He has a mobile phone number and he gives his sister’s phone number as next of kin. He has a Medicare card. Thomas is now staying in a refuge for the next month and is receiving support to access housing.

- ○ Highly vulnerable
- ○ Moderately vulnerable
- ○ Slightly vulnerable

Please provide the main rationale for your assessment decision

* 10. Please read the case study below, and based on your instinct of what you have read, assess the case study as either highly vulnerable, moderately vulnerable or slightly vulnerable.

Case 10 Summary

Clive is a 65 year old, who identifies as male and has severe dental pain. Clive has been living at Central Station for the past 5 years following the death of his partner, Samantha. Prior to that he was living in a rental apartment and working as a plumber. Clive hasn't seen a health professional for six years, and he finds it difficult to seek healthcare. He became distressed when living in the apartment alone and subsequently started street sleeping. Clive has a very quiet demeanour, his appearance is dishevelled, and he wears moderately soiled clothes. Clive has a slight tremor to his hands and upper body, he makes little eye contact, and tends to answer questions using only a few words. Clive denies any significant past medical or mental health history and tells you he has no mental health diagnosis and he isn't worried about his mental health. Clive appears withdrawn, and he has a depressed mood and appears moderately anxious. He denies any suicidal thoughts or thoughts of self-harm. He has no income and has been living from savings since he left the apartment. Clive denies having a support network. Clive has had pain to his teeth for over a year but didn't want to bother anyone. He is now having difficulty eating and so decided to seek some help. He denies any drug and alcohol use. Clive doesn't have a mobile phone, he doesn't have an emergency contact, but he can often be found sitting in Belmore Park during the day. Clive has a Medicare card.

- ◯ Highly vulnerable
- ◯ Moderately vulnerable
- ◯ Slightly vulnerable

Please provide the main rationale for your assessment decision

* 11. Please read the case study below, and based on your instinct of what you have read, assess the case study as either highly vulnerable, moderately vulnerable or slightly vulnerable.

Case 11 Summary

Darren, a 68 year old who identifies as male, and as an Aboriginal and was part of the stolen generation. Darren recently relocated from Darwin where he slept in the creek for many years. Darren has infected venous ulcers to both lower legs and is seeking to have them redressed. The wounds are bandaged, and the bandages are heavily soiled, as if they haven't been changed for over seven days. Darren has had the ulcers for several months, and he believes that they are getting worse. Darren was receiving regular dressings from a GP in Darwin but hasn't received any care since arriving in Sydney 2 days ago. He has been sleeping rough since leaving Darwin. Darren has long white hair and is unshaven, his clothes are moderately soiled with food and grime, and it appears as though he hasn't had a shower for several days. Darren says he last showered this morning. Darren believes that his wounds are related to his bad behaviour as an adolescent, that they are his penance. Darren appears to be experiencing auditory hallucinations. Darren denies any past medical or mental health history, although you notice he has a slight droop to his mouth and a weakness to his left arm, possible following a previous stroke. He states he doesn't take any medications. Darren denies any alcohol or drugs, and he denies any thoughts of suicide or self-harm, he is not worried about his mental health. He doesn't have a phone and no emergency contact. He does have a Medicare card. Darren has some money, but he doesn't say from where or whether he has any regular income.

- ○ Highly vulnerable
- ○ Moderately vulnerable
- ○ Slightly vulnerable

Please provide the main rationale for your assessment decision

* 12. Please read the case study below, and based on your instinct of what you have read, assess the case study as either highly vulnerable, moderately vulnerable or slightly vulnerable.

Case 12 Summary

Peter is a 40 year old who identifies as male, and he has been sleeping rough for the past six months since an alleged domestic and family violence incident with his wife. Peter has an alcohol dependence and has been drinking heavily for the past 3 years. He has had one litre of wine today. Peter was made redundant during COVID-19 in April 2020 and has been unable to gain further employment. He had all of his ID stolen a few months ago, and he currently has no income and begs for money in Sydney CBD. Peter has an unkempt appearance, his clothes are heavily soiled, and he is unshaven. He hasn’t had a wash in several months. Peter slurs his words and makes poor eye contact, he appears to have an extremely low mood and is not very engaging or forthcoming with information. Peter has some skin sores and some dental issues but he hasn't sought any healthcare. Peter denies any significant past medical history and isn’t on any medications, he hasn't seen a doctor for over a year. He denies any suicidal thoughts or thoughts of self-harm, but he is worried about his low mood. His next of kin is still is wife, and they haven’t spoken since she asked him to leave the house. Peter doesn’t have a Medicare card.

- ◯ Highly vulnerable
- ◯ Moderately vulnerable
- ◯ Slightly vulnerable

Please provide the main rationale for your assessment decision

* 13. Please read the case study below, and based on your instinct of what you have read, assess the case study as either highly vulnerable, moderately vulnerable or slightly vulnerable.

Case 13 Summary

Charles is a 70 year old, who identifies as male. He spends most of his days sleeping in doorways around Central Station, Sydney CBD. He is anorexic in appearance, and he is surrounded by different coloured plastic bags, food waste and general debris off the street. Charles is laying on his side and resting his head on the floor tiles of the doorway. Charles is trembling all over and he mumbles to himself repeatedly, as if he is experiencing auditory hallucinations. Charles hasn’t eaten food for days and is extremely dishevelled with heavily soiled clothes. Charles has kidney disease, and a history of depression. Charles refuses to take the medications prescribed for his depression. Charles intermittently seeks healthcare from the local emergency department. He was last admitted four months ago for a viral infection. In total, Charles has been sleeping on the streets for over 15 years. He believes that no one cares for him or what happens to him, he has no next of kin. He doesn’t have a phone, but he sleeps mostly in the Sydney CBD in shop doorways. Charles has a Medicare card and he receives income from Centrelink. He has no thoughts of suicide or self-harm. and he isn't worried about his mental health. He doesn’t drink alcohol or use illicit substances.

- ◯ Highly vulnerable
- ◯ Moderately vulnerable
- ◯ Slightly vulnerable

Please provide the main rationale for your assessment decision

* 14. Please read the case study below, and based on your instinct of what you have read, assess the case study as either highly vulnerable, moderately vulnerable or slightly vulnerable.

Case 14 Summary

Michael is a 56-year old who identifies as male. He has been sleeping rough since 2013. He has been alcohol dependent for several decades but has been sober for the past three months. He is at risk of death if he starts drinking again. Michael has short-term memory loss due to prolonged drinking and forgets where he is and who he is speaking to frequently. He has liver cirrhosis and COPD lung disease, which makes it difficult to walk long distances and to breathe generally. He has several respiratory inhalers which he takes regularly to help his breathing. He has a dishevelled appearance and hasn't showered in several weeks. In the last year he has been hospitalised every 3-months for a chest infection. He has multiple prior criminal convictions and has been in prison previously. He spent a few nights in custody last week for theft. He has attempted to get a job but been refused due to his offending history. Michael experienced past trauma, losing his mother at the age of 12, which is when he began street drinking and sleeping rough. He has also experienced trauma whilst street living, including having his stuff stolen and being attacked by members of the public. He is subsequently very fearful, and does not trust people easily. He also has trouble reading and writing so needs support to make applications. Michael has no ID and no bank account, no income, no Medicare card. He is difficult to track down due to memory problems and sleeps mostly in Sydney CBD. He has no emergency contact, but he is well known amongst others living on the streets in Sydney. He has no thoughts of self harm or suicide and is not worried about his mental health.

- ◯ Highly vulnerable
- ◯ Moderately vulnerable
- ◯ Slightly vulnerable

Please provide the main rationale for your assessment decision

* 15. Please read the case study below, and based on your instinct of what you have read, assess the case study as either highly vulnerable, moderately vulnerable or slightly vulnerable.

Case 15 Summary

Sam is a 36-year-old who identifies as female and was released from prison last week after four months of incarceration. Sam was unable to return to her parent's address or any other previous accommodation. She has been sleeping rough since her release. She had been in and out of prison over recent years due to minor theft charges and alleged assaults. She has experienced substance use problems and has had very little stability in her life. Sam has suffered from anxiety and depression for the past 10-years and has used cannabis and heroin to 'self-medicate'. She has recently been diagnosed with borderline personality disorder. She has asthma which she manages with inhalers. Sam smokes 10 cigarettes a day. She has no suicidal thoughts or thoughts of self-harm and is not worried about her mental health. A year ago she sought support from a drug and alcohol service and she is trying to stay clean as she is now six months pregnant. She started to smoke cannabis two nights ago due to increased anxiety. She appears anxious and is under the influence of cannabis. Sam has received support from housing agencies in the past, but this has been mainly temporary hostel accommodation and a short stay in supported housing. She has income from Centrelink. Sam is slightly dishevelled, she hasn't showered since leaving prison. She has a Medicare card and is able to seek healthcare when she needs to. She has a mobile phone and provides her parents as an emergency contact. She doesn't want to be pregnant and is fearful of living on the streets, because she is scared that other people will hurt her or her unborn child.

- ◯ Highly vulnerable
- ◯ Moderately vulnerable
- ◯ Slightly vulnerable

Please provide the main rationale for your assessment decision

* 16. Please read the case study below, and based on your instinct of what you have read, assess the case study as either highly vulnerable, moderately vulnerable or slightly vulnerable.

Case 16 Summary

JT is a 45 year old who identifies as non binary, originally from Germany and prefers to use the pronouns they/them. They were kicked out of home as a young teenager and started working as a chef. They moved to Australia in their early 20’s and became successful in running a catering business. They used drugs and alcohol to compensate for anxiety and depression and their addiction resulted in huge debts and a chaotic lifestyle that led to the bankruptcy of the business. They have lived on the streets for the past 15 years, intermittently staying in supportive housing. They have a tendency to hoard belongings and this has led to terminations of tenancy. They are currently street sleeping in Sydney CBD with their dog, Flash Gordon. They have no suicidal thoughts or thoughts of self-harm and they are not worried about their mental health. They have no consistent income. They have a Medicare card. Their past medical history includes hypertension, hepatitis C (untreated) and traumatic brain injury from previous assaults. Last admission to hospital was 6-months ago following an assault. They were prescribed an anti-hypertensive several years ago, but they don't take them very often. They have some difficulty concentrating and some short term memory problems, for which they recently sought review at a homeless health clinic and have been referred for a cognitive assessment and may soon be eligible for support from the National Disability Insurance Scheme. They are using heroin regularly and they appear slightly drowsy, with low mood. They don't drink alcohol. They have a dishevelled appearance, soiled clothes and they haven’t showered for several weeks. They have a mobile phone and an emergency contact.

- ○ Highly vulnerable
- ○ Moderately vulnerable
- ○ Slightly vulnerable

Please provide the main rationale for your assessment decision

* 17. Please read the case study below, and based on your instinct of what you have read, assess the case study as either highly vulnerable, moderately vulnerable or slightly vulnerable.

Case 17 Summary

Sylvia is a 27 year old who identifies as a transgender woman, she left her foster home at the age of 18 to live with a friend in Melbourne. She was diagnosed with borderline personality disorder and gender dysphoria at the age of 22 years. Sylvia has had difficulty holding down a job and she moved to Sydney three years ago to have a new start. Initially she was doing well, but then a relationship break-up led her to turn to alcohol and drugs. Sylvia hasn't been able to maintain work due to her impulsive behaviour, wild spending, and partying. She was subsequently evicted from her apartment a year ago for not paying the rent. Since her eviction, she regularly sleeps on the trains at night as she feels safe there. She isn't currently taking any medications and, she hasn't seen a health professional for two-years. She feels distrusting of the hospital system after several negative experiences with health professionals and is uncomfortable sitting in a waiting room. She drinks up to two litres of wine daily, and is slurring her words and having difficulty standing. She is tearful and appears anxious and hasn't showered or changed her clothes in several weeks. She has a Medicare card, no regular income and she sometimes provides sex for money. She avoids healthcare due to her past negative experiencines. She has a mobile phone but no emergency contact, she is estranged from her foster family. She has regular thoughts of suicide and self-harm and is worried about her mental health.

- ○ Highly vulnerable
- ○ Moderately vulnerable
- ○ Slightly vulnerable

Please provide the main rationale for your assessment decision

* 18. Please read the case study below, and based on your instinct of what you have read, assess the case study as either highly vulnerable, moderately vulnerable or slightly vulnerable.

Case 18 Summary

Brian is a 37 year old who identifies as male, living on the streets for two years. His daily routine is getting food from refuges, having a shower at Wayside Chapel, begging for money and then sleeping in squats or on the street. He used to own an apartment and was receiving a pension following a construction work related injury. He still receives a pension. He became addicted to cannabis and was evicted after not paying his rent. He has chronic back pain and reduced mobility to his right leg, which means he walks with a limp. He is otherwise generally well. He is prone to episodes of depression and anxiety and these usually correspond with the anniversary of his work accident. At the moment he is feeling very depressed and a month ago he self presented to hospital and spent a week as a hospital inpatient experiencing a major depressive episode. He has no thoughts of suicide, but has regular thoughts of self harm and is constantly worried about his mental health. He has been commenced on Escitalopram, which he takes as prescribed. He has a Medicare card, no next of kin, and he can be contacted on his mobile phone. He continues to smoke cannabis regularly and appears drowsy and has trouble concentrating.

- ○ Highly vulnerable
- ○ Moderately vulnerable
- ○ Slightly vulnerable

Please provide the main rationale for your assessment decision

19. Thank you for completing this survey, we really appreciate it. If you have any feedback regarding this survey, please leave your comments below.
