# Supplementary material for: Multistage Psychometric Testing of the Homeless Health Access to Care Tool
Source: Int J Environ Res Public Health. 2022 Nov 29;19(23):15928. doi: 10.3390/ijerph192315928 (PMC9738957; doi:10.3390/ijerph192315928)
Supplement: Supplementary file 1 [file ijerph-19-15928-s001.zip › File S2 HHACT Consumer Feedback.pdf]

Consumer Feedback on Completion of the Homeless Health Access to Care Tool

Thank you for answering the Homeless Health Access to Care Tool. I would like to ask for your feedback on your experience of answering the questions.

1. Did you find the questions in the Homeless Health Access to Care Tool easy to answer?

Y ☐ N ☐

Reasoning provided:

2. What did you think about the length of the survey?

Too short ☐ too long ☐ or just right ☐

Reasoning provided:

3. Do you think these questions are a good way to determine the health need and ability to access health care of a person experiencing homelessness?

Y ☐ N ☐

Reasoning provided:

4. Do you have any suggestions to improve the survey?

Y ☐ N ☐

Reasoning provided:
