# Supplementary material for: Multistage Psychometric Testing of the Homeless Health Access to Care Tool
Source: Int J Environ Res Public Health. 2022 Nov 29;19(23):15928. doi: 10.3390/ijerph192315928 (PMC9738957; doi:10.3390/ijerph192315928)
Supplement: Supplementary file 1 [file ijerph-19-15928-s001.zip › File S3 Interview Topic Guide.pdf]

Interview Topic Guide for Staff Involved in the administration of the HHACT

Introduction

Thank you for agreeing to participate in this interview regarding the administration of the HHACT to the Tierney House Participants. This process will take approximately 30 minutes and you are free to stop at any time. The interview will be led by Associate Professor Jane Currie. The researcher will use the questions below as a guide and will allow the conversation to flow freely. The interview will be audio recorded with your permission to facilitate transcription.

Please let me know if you have any questions. If there aren't any questions then the recording will begin now.

1. How did you feel administering the Homeless Health Access to Care Tool?
2. Were there any questions that felt awkward or clunky to ask?
3. How did you feel about the order of the questions in the Homeless Health Access to Care Tool? Did the questions fit together nicely?
4. Did you feel as if you were repeating yourself at any stage?
5. Did you think that you missed anything important that you would normally find out during a consultation?
6. What did you think about the length of the Homeless Health Access to Care Tool? Were the participants able to stay engaged for that period?
7. How did it compare to administering the VI-SPDAT?
8. How comfortable did you feel asking the questions in the Homeless Health Access to Care Tool?
9. Did any of the questions cause the participants to become distressed or create tension?
10. Were there any questions that you felt like you couldn't or shouldn't ask?
11. Any further comments?

The recording will be stopped now.

The interview is now over, thank you for participating. Your contribution is greatly appreciated.
